# Supplementary material for: T cells specific for post-translational modifications escape intrathymic tolerance induction
Source: Nat Commun. 2018 Jan 24;9:353. doi: 10.1038/s41467-017-02763-y (PMC5783942; doi:10.1038/s41467-017-02763-y)
Supplement: Supplementary file 1 — Supplementary Information [file 41467_2017_2763_MOESM1_ESM.pdf]

Supplementary figure legends

a

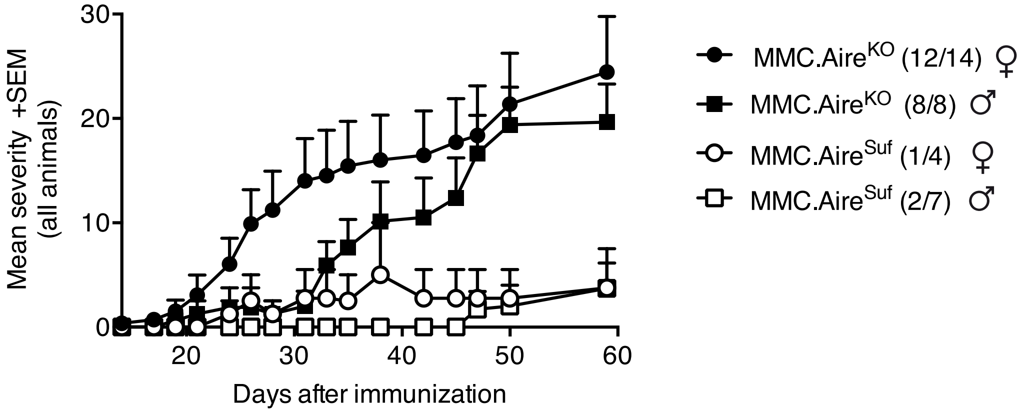

b

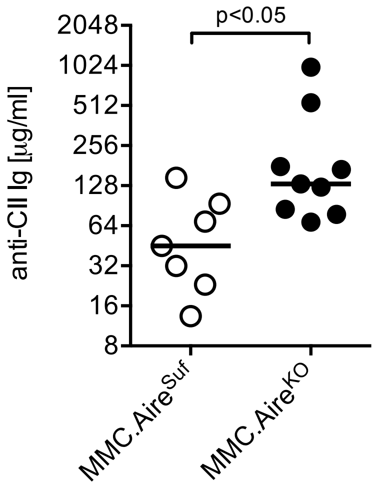

Supplementary figure 1 – Aire deficiency increases disease susceptibility and antigen-specific antibody titers in MMC mice.

a) Mean severity of arthritis following immunization with CII in CFA. Both healthy and arthritic animals are represented, and number in brackets shows the number of arthritic animals over the total number of animals included in the group. Error bar shows SEM. b) Autoantibody levels five weeks after immunization. Lines indicate median value. p-value was calculated using Mann-Whitney U test. The titers of total anti-CII antibodies were determined by ELISA[1] †. Briefly, serum was titrated (1:10 to 1:10^6) in parallel to the

standard and titer values were interpolated within the linear range and related to the standard curve. Biotinylated rat anti-mouse Ig каppa (clone 187.1; our collection) was used as detecting antibody. Binding of biotinylated antibodies was done with extravidin peroxidase (Sigma-Aldrich). Plates were developed using ABTS (Roche Diagnostics, Mannheim, Germany) as substrate, and the absorbance was measured at 405 nm (Synergy-2; BioTek, Winooski, VT, USA). Total anti-CII Ig levels were measured (µg/ml) using purified polyclonal anti-CII IgG antibodies of a known concentration as a standard.

Reference:

1. Holmdahl, R., Klareskog, L., Andersson, M. & Hansen, C. High antibody response to autologous type II collagen is restricted to H-2^{q}. *Immunogenetics* **24,** 84–89 (1986).

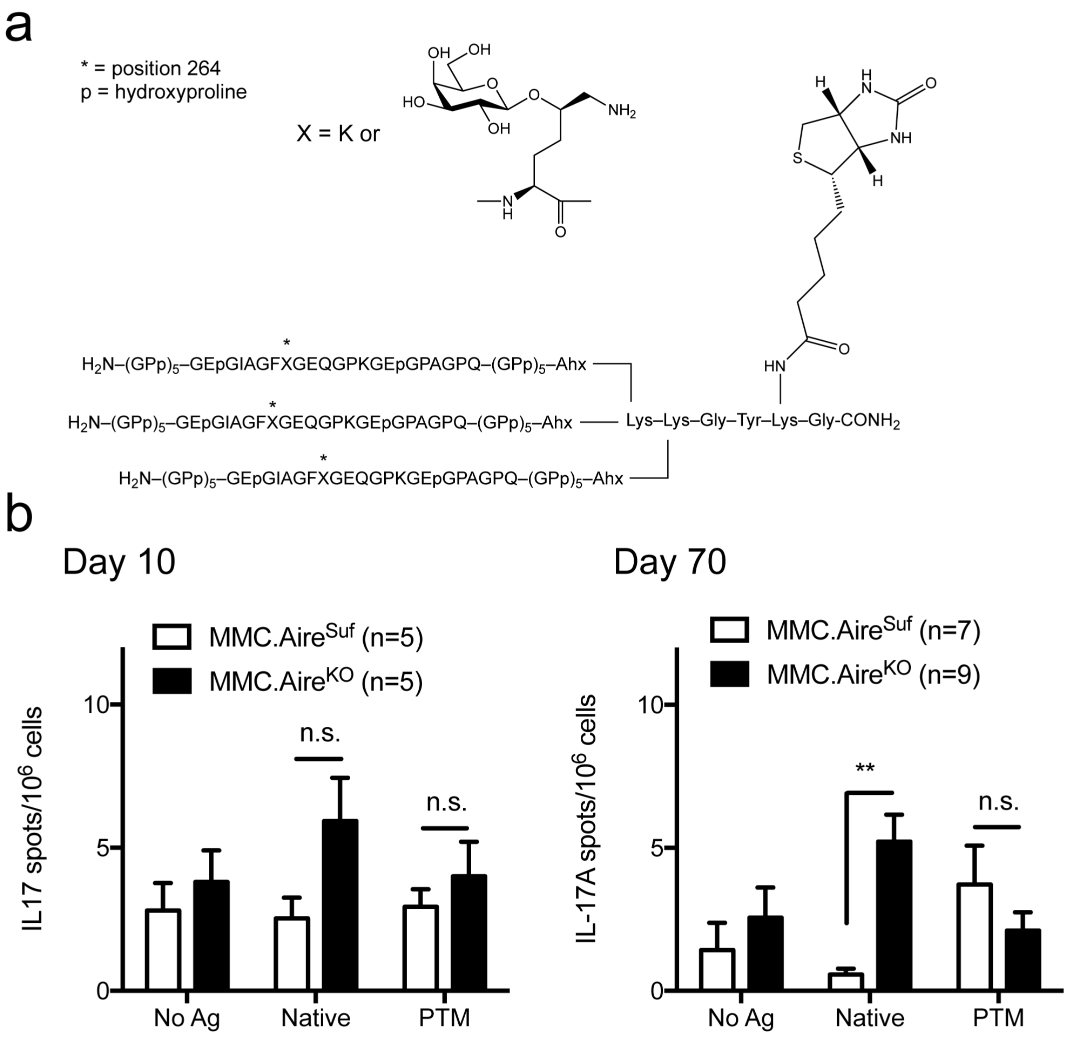

Supplementary figure 2 – Structure of the triple helical CII peptides and IL-17A response to each of the peptides in Aire sufficient and Aire deficient MMC mice. (a) Chemical structure of the triple helical CII peptides. (b) *In vitro* recall responses of pooled lymph node and spleen cells from indicated number of MMC.Aire^{Suf} and MMC.Aire^{KO} mice. Organs were collected 10 days or 10 weeks after immunization with rat CII in CFA. Cells were stimulated with the non-modified CII peptide (native) or with the galactosylated CII peptide (PTM) or left unstimulated (No Ag). Values shown are the mean ± SEM number of spots

recorded for IL-17A producing cells. n.s., not-significant; ** $p<0.01$ (Mann-Whitney U test).

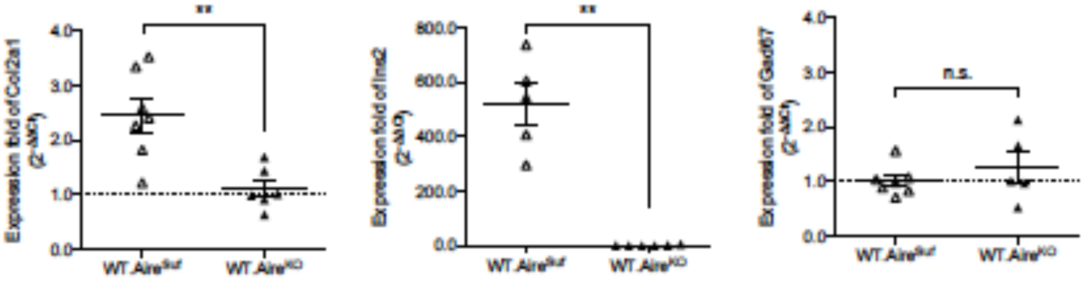

Supplementary figure 3 – Thymic expression of the CII$_{260\text{-}270}$ epitope in WT mice.

The mouse CII$_{260\text{-}270}$ epitope is expressed in the thymus of non-MMC mice (WT) in an Aire-dependent fashion. cDNA derived from thymi of WT.Aire^{Suf} (n=7) and WT.Aire^{KO} (n=6) mice was prepared and quantitative PCR was used to determine gene expression of CII, using gene-specific labeled probes. Aire-dependent (*Ins2*) and -independent (*Gad67*) genes were used as controls. Data was normalized to the expression of cyclophilin A (*Ppia*) and calibrated with a WT.Aire^{KO} sample. Lines indicate mean value ± SEM and p-values were calculated using Mann-Whitney U test. **, $p<0.01$; n.s., not significant.

Supplementary figure 4

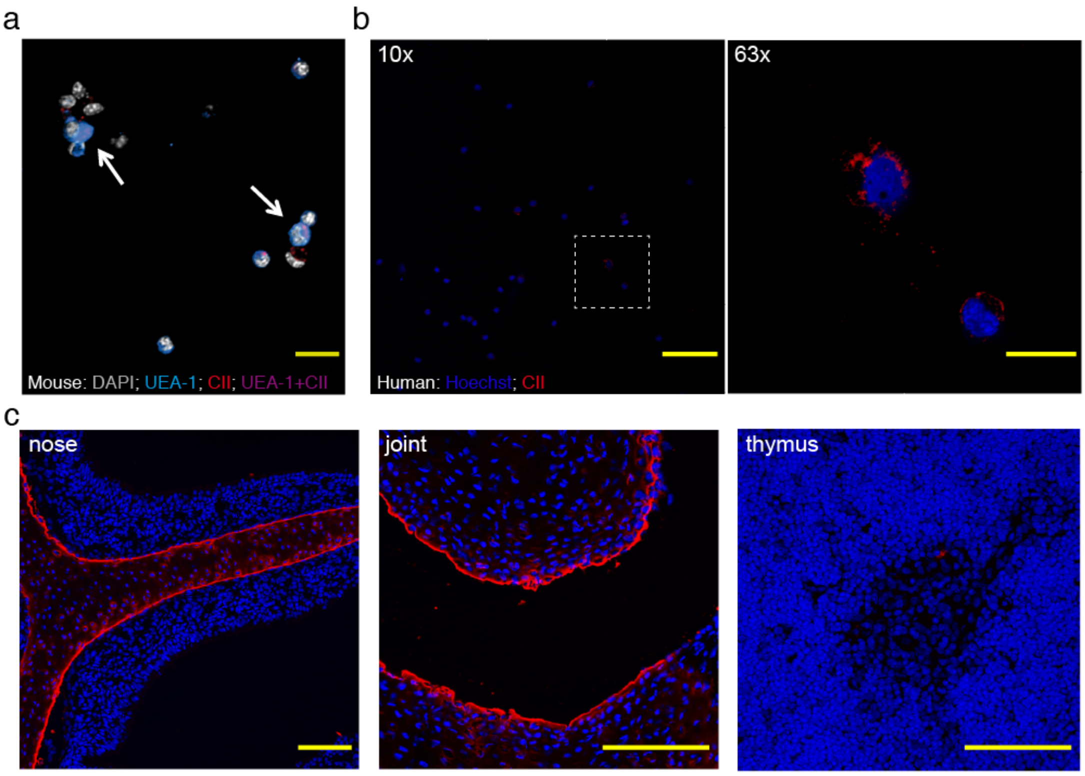

Supplementary figure 4 – PTM CII is differently accessible in the joint and thymus. (a) Enriched murine thymic epithelial cells were stained for DNA (DAPI, in gray), mTEC (UEA, in blue) and CII (in red). White arrows indicate cells positive for both UEA-1 and CII (in purple). Scale bar indicates 20 μm (b) Cytospin of sorted human thymic epithelial cells as defined by $CD45^-$ $EpCAM^+CDR2^-HLA\text{-}DR^{hi}$ stained with anti-CII mAbs in red and nucleus in blue (Hoechst). Approximately 5-10% of the $HLA\text{-}DR^{hi}$ cells stained positive for CII. No CII-stain was observed in $HLA\text{-}DR^{lo}$ cells. Scale bar indicates 100 μm and 20 μm in the left and right panels, respectively. (c) Nasal and joint cartilage and thymus from WT mouse stained with Hoechst (blue) and CII (mAb cocktail, in red). Scale bar indicate 100 μm.

Supplementary Fig. 5

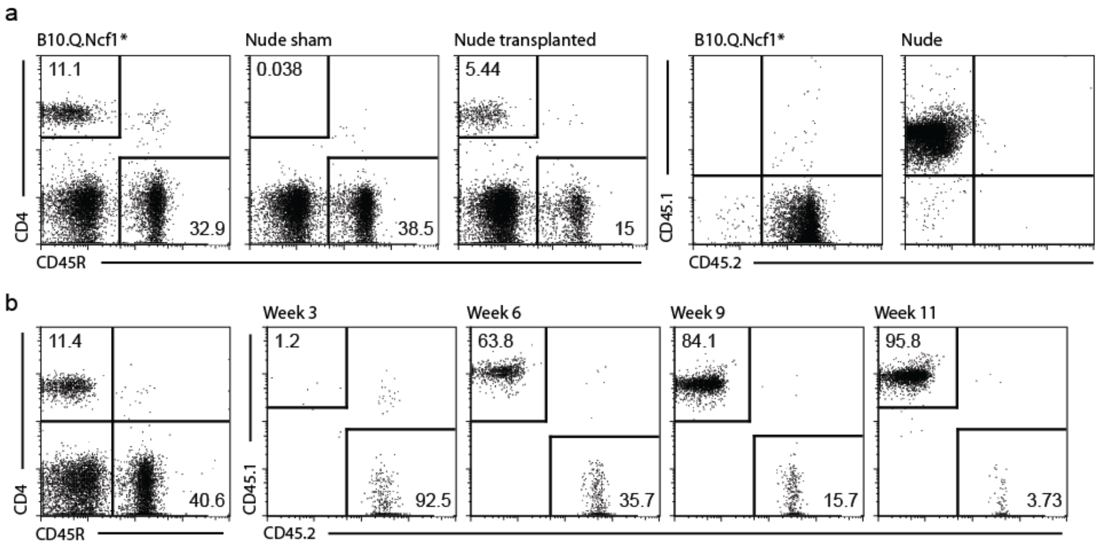

Supplementary figure 5 - Establishment of a peripheral T cell pool in athymic nude mice following thymus transplantation.

Establishment of a peripheral T cell pool in athymic nude mice occurs within 11 weeks after thymus transplantation. a) Thymi from newborn WT or MMC donor mice (CD45.2) were transplanted into four weeks old nude mice (CD45.1), resulting in the appearance of $CD4^{+}CD45.2^{+}$ T cells in the blood of recipient mice some weeks later. b) More than 95% of the circulating $CD4^{+}$ T cells in the blood of transplanted nude mice were of recipient origin ($CD45.1^{+}$) 11 weeks after thymus transplantation.

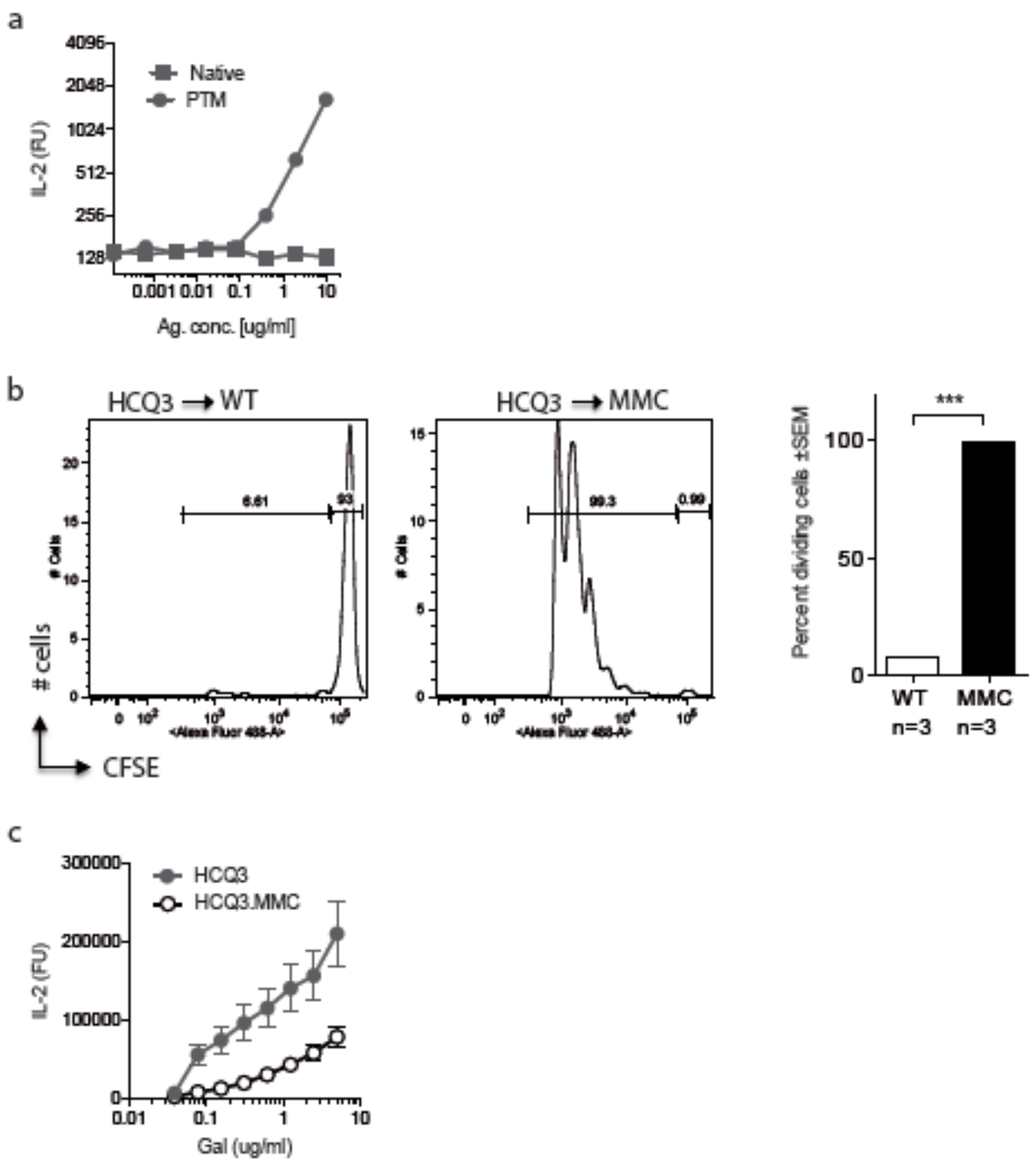

Supplementary figure 6 - T cell specificity and responses in HCQ3 TCR transgenic mice.

a) IL-2 production of spleen cells from naïve HCQ3 transgenic mice after *in vitro* stimulation with titrated amounts of the naïve or PTM variant of the $CII_{260\text{-}270}$ peptide. b) Adoptive transfer of CFSE-labeled T cells from naïve HCQ3-transgenic mice on the RAG1-deficient background into naïve WT or MMC recipients. T cell activation was determined by dilution of CFSE in transferred T cells harvested from inguinal lymph nodes 3 days later. Right panel shows the mean number of

dividing cells for three recipients per group. c) *In vitro* recall response of pooled spleen and lymph node cells to titrated amounts of PTM $CII_{260-270}$ peptide 10 days after immunization of HCQ.3 (n=6) and HCQ3.MMC (n=5) mice with rat CII in CFA.

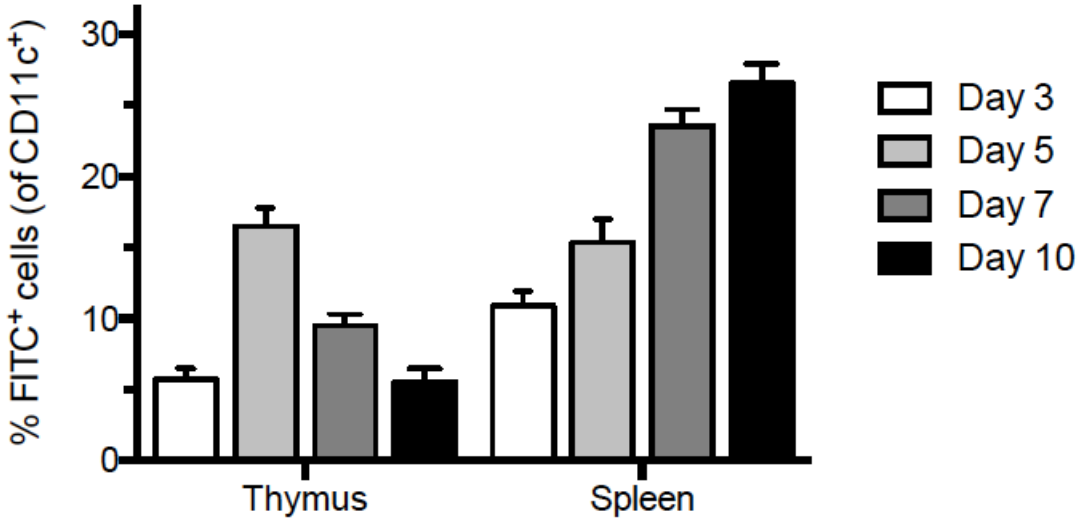

Supplementary figure 7 - Peripheral antigen migrates to the thymus and can be found in CD11c+ cells.

200µl of 2.5% solution of YG beads were injected s.c. in the flank of WT mice. Thymus and spleen were collected 3, 5, 7 and 10 days later, and analyzed for the presence of FITC+ cells (green channel). FITC+ cells were exclusively CD11c+ and accounted for >15% of all analyzed CD11c+ cells, 5 days after s.c. injection.

Supplementary table 1 – Summary of staining of tissue and cells using CII-specific antibodies. Human thymus and murine thymus and joints were stained with different conformation-specific anti-CII mAb and assessed by microscopy. Frequency of CII^{+} cells in mTECs (cytospin of enriched thymic epithelial cells) or medulary regions of from sections was assessed.

**Murine cartilage**	**mAb cocktail**	**PTM (clone T8)**	**M2139-S31R**
WT (n=3)	all positive	all positive	all negative
MMC (n=4)	all positive	all positive	all negative

**Murine thymus**	**mAb cocktail**	**PTM (clone T8)**	**M2139-S31R**	**CII^{+} per medula**
WT (n=3)	all positive	all negative	all negative	4.5 ± 1.6
MMC (n=4)	all positive	all negative	all negative	6.3 ± 2.3

**Human thymus**	**CII^{+} HLA-DR^{hi}**	**CII^{+} HLA-DR^{lo}**	**$CII^{+}Aire^{+}$ per medula**
mAb cocktail	43/763 (n=1)	0/775 (n=1)	1.5 ± 0.5 (n=2)
M2139	n/a	n/a	1.3 ± 0.7 (n=3)
M2139-S31R	n/a	n/a	0.0 (n=2)
